# Performance evaluation and clinical validation of optimized nucleotide MALDI-TOF-MS for mycobacterial identification

**DOI:** 10.3389/fcimb.2022.1079184

**Published:** 2022-12-02

**Authors:** Baiying Li, Chi Zhu, Lifang Sun, Hang Dong, Yaping Sun, Shangzhi Cao, Libo Zhen, Qi Qi, Quanquan Zhang, Ting Mo, Huijie Wang, Meihua Qiu, Chao Song, Qingshan Cai

**Affiliations:** ^1^ Department of Tuberculosis, Affiliated Hangzhou Chest Hospital, Zhejiang University School of Medicine, Hangzhou, China; ^2^ State Key Laboratory of Translational Medicine and Innovative Drug Development, Jiangsu Simcere Diagnostics Co., Ltd., Nanjing, China

**Keywords:** nucleotide MALDI-TOF-MS, mycobacterium, tuberculosis, NTM, diagnostic efficiency

## Abstract

**Objective:**

To evaluate the performance and validate the diagnostic value of a nucleotide matrix-assisted laser desorption time-of-flight mass spectrometry (MALDI-TOF-MS) with the analysis process optimized in identification of mycobacterium species.

**Methods:**

The optimized analysis process was used for mycobacterial identification in the nucleic MALDI-TOF-MS. 108 samples were used for assessing the performance of nucleic MALDI-TOF-MS, including 25 reference standards, 37 clinical isolates, 37 BALF, and 9 plasmids. The BALF of 38 patients suspected of pulmonary mycobacterial infection was collected for validation. Clinical etiological diagnosis was used as the gold standard to evaluate the diagnostic value of nucleotide MALDI-TOF-MS.

**Results:**

The sensitivity, specificity, and accuracy of the nucleotide MALDI-TOF-MS in mycobacterial identification were 96.91%, 100% and 97.22%, respectively, and the limit of detection for mycobacterium tuberculosis (MTB) was 50 bacteria/mL. Among 38 patients suspected of pulmonary mycobacterial infection, 33 were diagnosed with pulmonary tuberculosis infection, and 5 with non-mycobacterial infection. In clinical validation, the positive rates of MALDI-TOF-MS, Xpert MTB/RIF, culture and AFS in BALF of patients diagnosed with tuberculosis infection were 72.7%, 63.6%, 54.5% and 27.3%, respectively. The sensitivity/specificity of MALDI-TOF-MS, Xpert, culture and AFS in diagnosing MTB were 72.7%/100%, 63.6%/100%, 54.5%/100%, 27.3%/100%, with the areas under the curve of 0.864, 0.818, 0.773, and 0.636, respectively.

**Conclusion:**

Optimized nucleotide MALDI-TOF-MS has satisfactory sensitivity, specificity and low LOD in the identification of mycobacteria, which may serve as a potential assay for mycobacterial identification.

## 1 Introduction

Tuberculosis (TB) has long been considered one of the world’s leading causes of death. In 2020, 59% of the global TB cases were confirmed by bacteriological tests. So far, however, about 4.1 million TB patients worldwide have not been diagnosed or reported, and World Health Organization (WHO) modelling projections suggest that the number of TB patients and deaths could be even higher in 2021 and 2022 (Jeremiah et al., 2022). Non-tuberculous mycobacterium (NTM) refers to a general category of mycobacterium except mycobacterium tuberculosis (MTB) complex and mycobacterium leprae. In recent years, there has been an increasing trend of NTM infection. NTM can be temporarily, intermittently or long-term colonization in the lungs of the human body without causing disease, which results in considerable difficulties in deciding which patients to treat and when to treat. In addition, diseases caused by NTM have various characteristics, antimicrobial resistance spectrum, and treatment schemes. Identification of NTM species can help clinical diagnosis and treatment of NTM. Therefore, research and development of innovative technologies for MTB/NTM diagnosis is imminent.

Traditionally, identification of MTB depends on isolates and culture in liquid or solid medium in biosafety level 2/3 laboratory, which has a long culture period and low positive rate. Advances in high-throughput sequencing and bioinformatics technologies have led to a rapid increase in the application of metagenomics in pathogenic detection (Wilson et al., 2014; Simner et al., 2018; Armstrong et al., 2019; Blauwkamp et al., 2019; Ye et al., 2019). The newly revised Chinese health industry standard “Tuberculosis Diagnosis WS 288-2017” has added to the results of molecular biology in the confirmed diagnosis of TB, indicating that molecular biology technology will play an increasingly important role in the diagnosis and treatment of TB. The currently available molecular biological methods for rapid detection of MTB include Gene Chip (Wu et al., 2018), targeted NGS (tNGS) (Kambli et al., 2021), Xpert MTB/RIF (Xpert) (Bunsow et al., 2014), Loop-mediated isothermal amplification (LAMP) (Detjen et al., 2015), PCR (Shen et al., 2020), and more. Although these techniques can be used to detect clinical specimens directly, there are also some limitations, such as only detection of drug resistance genotypes but not bacterial identification and verification, simultaneous bacterial identification and resistance to a certain drug, identification of MTB but not NTM, time-consuming and costly. Nucleic MALDI-TOF-MS detection integrates the high sensitivity of PCR technology, high throughput of chip technology, and high precision of MALDI-TOF-MS. One reaction system can achieve multiple gene amplifications used to analyze single nucleotide polymorphisms, gene mutations, DNA methylation and copy number identification (Kang et al., 2018). The chip can be used in batches, with good expansibility. Single hole can realize 10-40 retesting, processing up to 3000 samples per day. In addition, manual operation is less than 30 minutes, the test results can be issued within 8 h at the earliest. Because the detection does not require the fluorescent probe, the overall cost of detection is low. In general, the detection efficiency and throughput of this technique are much higher than that of fluorescence quantitative PCR, and the detection cycle is much lower than that of the first- and second-generation sequencing (≥18 h) (Trembizki et al., 2014; Kriegsmann et al., 2015), so it has a broad application prospect in clinical practice.

There are many reports on the identification of mycobacteria based on protein MALDI-TOF-MS (Body et al., 2018; Luo et al., 2018; Rodriguez-Temporal et al., 2018; Rodriguez-Temporal et al., 2020; Fernández-Esgueva et al., 2021). However, the application of nucleotide MALDI-TOF-MS technology in the identification of mycobacterium is rarely reported. The aim of this study was to evaluate the performance of nucleotide MALDI-TOF-MS in mycobacterial identification and its real-world application.

## 2 Materials and methods

### 2.1 Study design and sample source

Reference standards, clinical isolates, bronchoalveolar lavage fluid (BALF), and plasmids were all included in performance evaluation. qPCR and next-generation metagenomic sequencing (mNGS) were used to identify the species in BALF for MALDI-TOF-MS validation. The validation results were used as the gold standard to evaluate the performance of nucleotide MALDI-TOF-MS. The limit of detection (LOD) of nucleotide MALDI-TOF-MS was evaluated according to the national reference standards for PCR-based detection of MTB from National Institutes for Food and Drug Control (China). The LOD was determined by the lowest detected reference (S1-S4) repeated five times. S1, S2, S3 and S4 were single-cell suspensions of MTB (CMCC 93009), and the corresponding concentrations were 1×10^3^ bacteria/mL, 2×10^2^ bacteria/mL, 1×10^2^ bacteria/mL, 50 bacteria/mL and 25 bacteria/mL, respectively.

The BALF of patients suspected of pulmonary mycobacterial infection in the Department of Tuberculosis, Affiliated Hangzhou Chest Hospital was collected prior to antituberculosis therapy and submitted for nucleotide MALDI-TOF-MS, along with acid-fast staining (AFS) (Wang et al., 2018), culture (Wang et al., 2018), Xpert MTB/RIF (Bunsow et al., 2014) and other experiments. These results of diagnostic methods were all obtained from the medical records of patients in the clinical service. Clinical etiological diagnosis was used as the gold standard to evaluate the diagnostic value of nucleotide MALDI-TOF-MS.

### 2.2 mNGS detection

A micro-sample genomic DNA extraction kit (DP316, Tiangen) was used to extract the nucleic acid. NEBNext Ultra II DNA Library Prep Kit (New England Biolabs Inc.) was used to construct Illumina sequencing libraries and Nextseq 550 DX (75 bp single-end reads; Illumina) was used for sequencing. An alignment tool (Burrows-Wheeler Alignment) was used to map to a human reference genome (GRCh38) to exclude human sequence data. The remaining sequencing data were aligned to NCBI nt database by SNAP. The specific detection method of mNGS can be referred to our previous report (Zhang et al., 2022).

### 2.3 Nucleotide MALDI-TOF-MS detection

Through the nucleotide MALDI-TOF-MS detection, various mycobacterial species including MTBC and 23 kinds of NTM can be identified. Details were shown in **Table 1**.

**Table 1:** The identification catalog of mycobacterial species.

NO.	Mycobacterial species	NO	Mycobacterial species
1	MTBC	13	M. scrofulaceum
2	M. kansasii	14	M. marinum
3	M. abscessus	15	M. gastri
4	M. chelonae	16	M. intracellulare
5	M.celatum	17	M. simiae
6	M. shimoidei	18	M. terrae
7	M. smegmatis	19	M. peregrinum
8	M. avium complex	20	M. gordonae
9	M. fortuitum	21	M. genavense
10	M. asiaticum	22	M. septicum
11	M. ulcerans	23	M. chimaera
12	M. xenopi	24	M. massiliense

MTBC, Mycobacterium tuberculosis complex.

#### 2.3.1 DNA extraction

The samples were extracted according to the instructions of MagPure DNA Kit. 40 µL proteinase K mixed with 0.5 mL samples and 50 µL buffer SDS into a 2 mL homogenate tub. After inverted mixing, the homogenizer was shaken for 2 minutes, and then incubated at 60°C for 10 min and centrifuged at 10 000 x g for 3 min. 500 µL of digestive solution was transferred to a new deep-well plate. The corresponding program was implemented after each deep-well plate was placed correctly in the corresponding position of the instrument. The DNA obtained was stored at -20°C after running the extraction procedure on the MagPure automatic extractor.

#### 2.3.2 PCR reaction

The PCR reaction consisted of 1 μL10×PCR buffer with 25 mM MgCl_2_, 0.8 μL 25 mM MgCl_2_, 0.1 μL UNG (heat labile), 0.2 μL dUTP/dNTP Mix, 0.4 μL PCR enzyme, and 1 μL DNA template. During operation, the DNA was diluted to 10 ng/μL, 6.5 uL DNA was added to the PCR system. The PCR reaction was performed as follows: 25°C for 5 minutes, 95°C for 2 minutes, 45 cycles at 95°C for 30 seconds, at 56°C for 30 seconds, at 72°C for 60 seconds and final extension at 72°C for 5 minutes.

#### 2.3.3 SAP reaction

SAP mix was prepared into a final volume of 4 μL (3.06 μL RNase-free water, 0.34 μL SAP buffer and 0.60 μL SAP enzyme), then added to the PCR and incubated for 40 minutes at 37°C, finally ended with 5 minutes of inactivation at 85°C.

#### 2.3.4 Extension reaction

A 4.0 μL iPLEX extension reaction containing 1.24 μL RNase-free water, 0.40 μL iPLEX buffer plus (10×), 0.40 μL iPLEX termination mix, 0.08 μL iPLEX pro enzyme and 1.88 μL iPLEX pus extend primer mix were added to each well. The extension reaction was performed in the following steps: denaturation at 95°C for 30 seconds, followed by 40 cycles of 94°C for 5 seconds, five rounds of annealing at 52°C for 5 seconds, and extension at 80°C for 5 seconds, then final extension at 72°C for 3 minutes. The information of genes and primers designed for TB detection is shown in **Table 2**.

**Table 2:** TB primer sequence and single base extension primer.

Target gene	Primer	Primer sequence(5’-3’)	Amplified products(bp)	Single nucleotide extension	Relative molecular mass of extension primer	Extension base	Relative molecular mass of extension product	Gene reference sequence
IS1081	IS1081_F	ACGTTGGATGCGTCGAGTACCCGATCATAT	90	TTGGGCAACAACTGA	4602	A	4929.1	CCTGCTGCACTCCATCTACgaccagcccgacgccga[A/G]tcagttgttgcccaATATGATCGGGTACTCGACG
	IS1081_R	ACGTTGGATGCTGCTGCACTCCATCTACGA						
IS6110	IS6110_F	ACGTTGGATGTCCAGCGCCGCTTCGGACCA	131	GACCTCACCTATGTGTC	5122.3	G	5409.5	CCTGCGAGCGTAGGCGTCGGtgacaaaggccacgtaggcgaaccctgcccaggt[A/G]gacacataggtgaggtctgctacccacagccggttaggtgctggtggtCCGAAGCGGCGCTGGACGAG
	IS6110_R	ACGTTGGATGCGTAGGCGTCGGTGACAAA						

F, forward primer; R, reverse primer.

#### 2.3.5 Sample desalting

10 μL of water was added to each well of the reaction plate, and the automatic desalting procedure was performed using the MASSARRAY^®^ instrument.

#### 2.3.6 Mass spectrometry analysis

Mass spectrometry data of the samples were obtained by MassARRAY^®^ Typer, and bioinformatics was analyzed using the self-developed bioinformatics pipeline based on PYTHON 3. The positive samples were determined by calculating the extension rate of the assay site. The assay with an extension rate greater than the set threshold would be judged as positive, while that near the threshold would be in the grey area of analysis. The sites located in the gray area were manually interpreted and analyzed.

### 2.4 Statistical analysis

SPSS 22.0 statistical software was used for data analysis, and Graphpad Prism 8 and R were used for plotting. Non-normally distributed data were expressed as the median [first quartile (Q1), third quartile (Q3)], and non-parametric Mann-Whitney U test was used for comparison between groups. The counting data were expressed as the number of cases (percentage) [n (%)], and the data between groups were compared by chi-square test or Fisher’s exact test. 2×2 contingency tables and receiver operating characteristic (ROC) curves were used to evaluate the diagnostic efficacy. A two-tailed value of *p*<0.05 represented significant differences.

## 3 Results

### 3.1 Sample selection and characteristics

**Figure 1:**
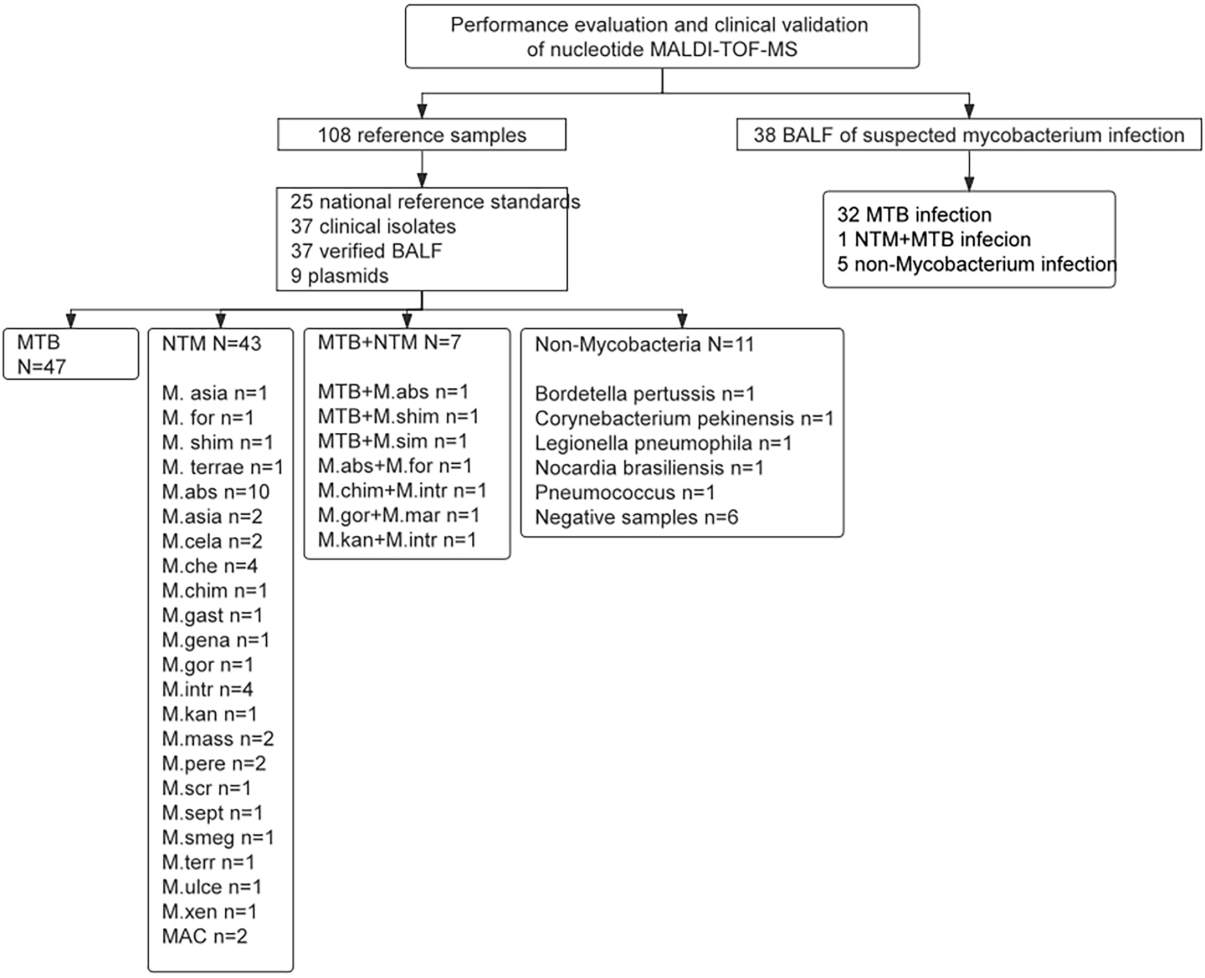
The selection diagram of samples for performance evaluation and clinical validation. MTB, Mycobacterium tuberculosis; NTM, Nontuberculous mycobacteria; MAC, Mycobacterium avium complex.

A total of 108 samples were used for performance evaluation, containing 25 national reference standards, 37 clinical isolates, 37 verified BALF, and 9 plasmids, which covered 24 types of mycobacteria and other non-mycobacteria. From March to June 2022, a total of 40 clinical samples (BALF) were collected from patients with suspected pulmonary mycobacterial infection in the Department of Tuberculosis, Affiliated Hangzhou Chest Hospital for clinical validation. The sample data of metagenomic validation results are in **Supplementary Table 1**. Two samples were excluded due to missing clinical information. The selection diagram of samples for performance evaluation and clinical validation is shown in **Figure 1**.

Among 38 samples eligible for clinical validation, there were 22 males and 16 females, with the median age of 36 years. 34.2% of patients had pulmonary shadow by physical examination, and 44.7% experienced cough. Both lung lobes were involved in 68.4% of patients (**Table 3**). 86.8% were eventually diagnosed with pulmonary TB infection, among whom 1 case was diagnosed with a mixed infection of MTB and *mycobacterium cheloniae.*

**Table 3:** Characteristics of 38 patients used for clinical validation.

Characteristics	Data
Age, years, median (Q1, Q3)	36 (26,64)
Gender, male, n (%)	22 (57.9)
Pulmonary shadow discovered by physical examination	13 (34.2)
Underlying disease, n (%)	
Hypertension	3 (7.9)
Diabetes	5 (13.2)
Renal insufficiency	2 (5.3)
Comorbidities, n (%)	
Cough	17 (44.7)
Chest distress and (or) chest pain	6 (15.8)
Fever	6 (15.8)
Discharged diagnosis, n (%)	
Pulmonary tuberculosis infection	33 (86.8)
Pulmonary infection (Non-TB/NTM)	5 (13.2)
Pulmonary lobe involvement, n (%)	
Both lung lobe	26 (68.4)
Right superior lobe	4 (10.5)
Left superior lobe	6 (15.8)
Right lobe	2 (5.3)

TB, Tuberculosis; NTM, Nontuberculous mycobacteria.

### 3.2 Performance evaluation

**Figure 2:**
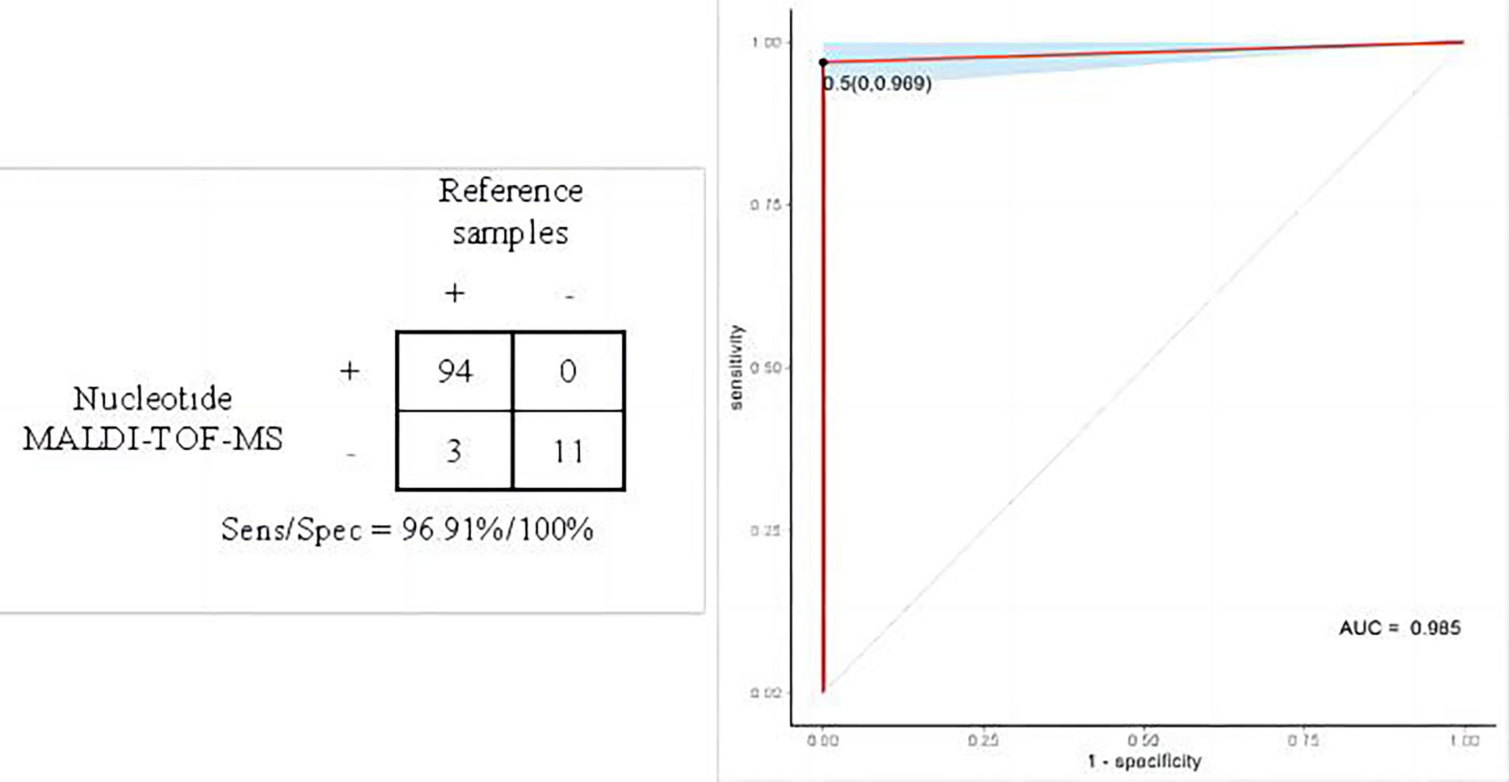
Performance evaluation of nucleotide MALDI-TOF-MS in the identification of mycobacterial species.

105 of the 108 results of nucleotide MALDI-TOF-MS was consistent with reference samples. The remaining 3 cases of *M. abs* were negative in BALF samples. The positive results of MALDI-TOF-MS detection are shown in **Supplementary Table 2**. The sensitivity, specificity and accuracy of nucleotide MALDI-TOF-MS in the identification of mycobacterial species were 96.91%, 100% and 97.22%, respectively, with the area under the curve of 0.99 (**Figure 2**), and 1×10^3^ bacteria/mL, 2×10^2^ bacteria/mL, 1×10^2^ bacteria/mL, 50 bacteria/mL could be detected stably. Notably, the result was negative when the concentration of MTB was diluted to 25 bacteria/mL. The LOD of MALDI-TOF-MS for MTB was 50 bacteria/mL.

### 3.3 MTB detected by multiple methods

**Figure 3:**
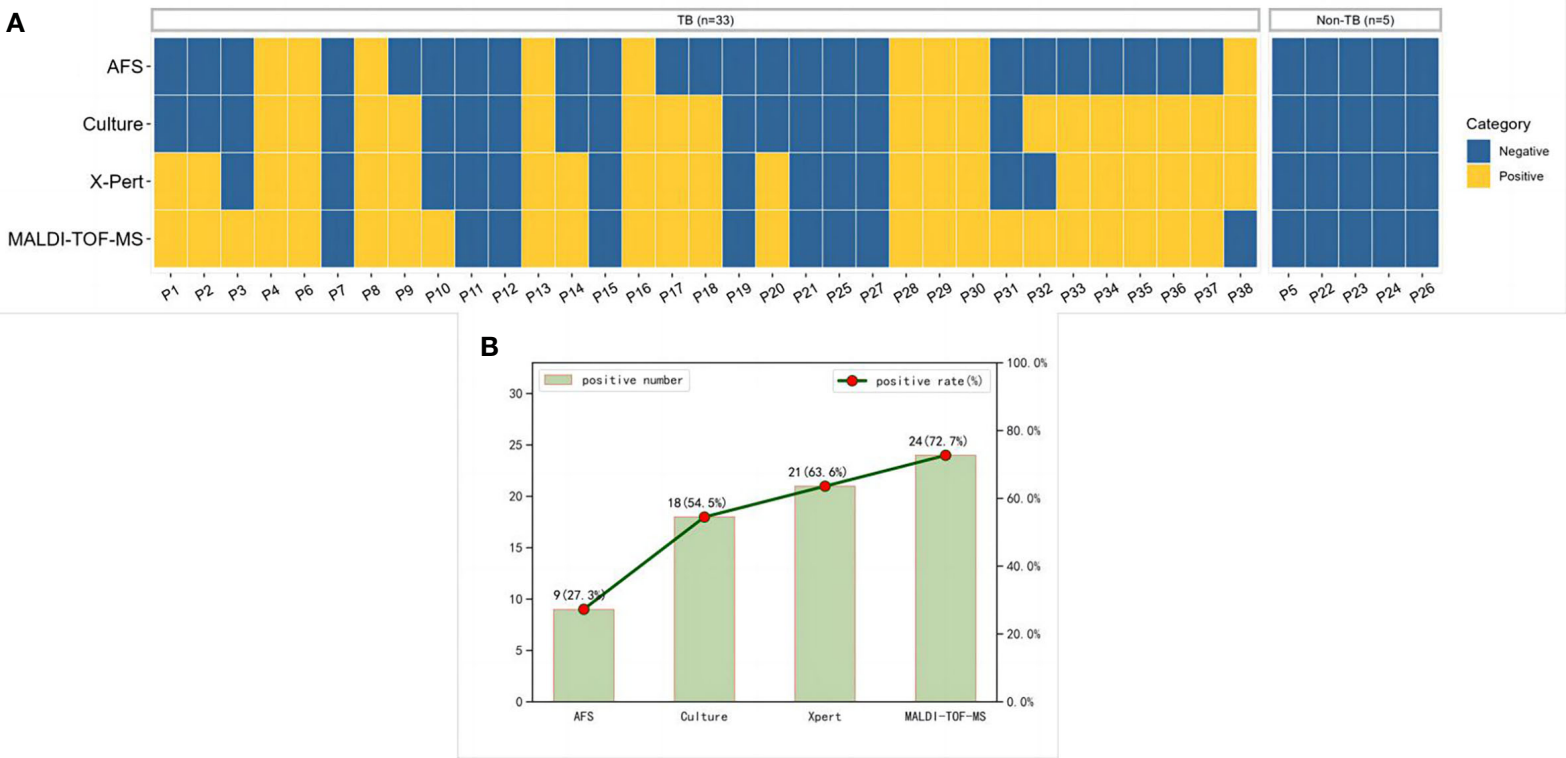
MTB results detected by multiple methods. **(A)** A heatmap depicting the identification of MTB in BALF samples by different methods. **(B)** Positive rates of each method in patients diagnosed with pulmonary tuberculosis. MTB, Mycobacterium tuberculosis; BALF, Bronchoalveolar lavage fluid.

The specific detection results of AFS, culture, Xpert, and MALDI-TOF-MS in BALF of 33 patients diagnosed with TB and 5 patients with non-TB infection were shown in **Figure 3A**. The positive rates of MALDI-TOF-MS, Xpert, culture and AFS in BALF of patients diagnosed with TB infection were 72.7%, 63.6%, 54.5% and 27.3%, respectively (**Figure 3B**).

### 3.4 Clinical validation of MALDI-TOF-MS in diagnosing MTB

**Figure 4:**
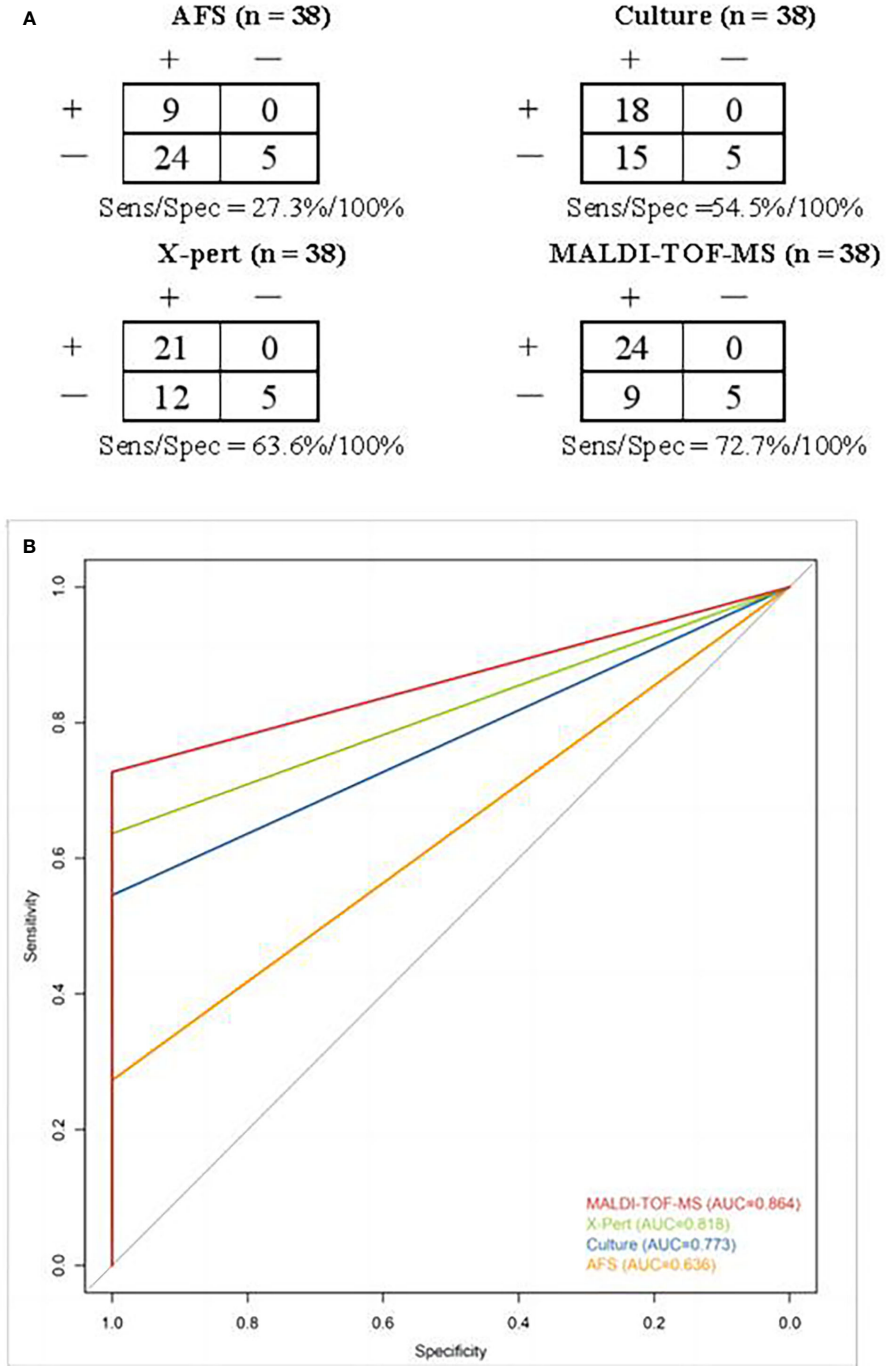
**(A)** Contingency tables for different methods in detecting MTB. Clinical discharge diagnosis was used as a reference method. **(B)** ROC curves and areas under the curve of different methods in diagnosing MTB. ROC, Receiver operator characteristics; MTB, Mycobacterium tuberculosis.

As shown in **Figure 4A**, the sensitivity/specificity of MALDI-TOF-MS, Xpert, culture and AFS in the diagnosis of MTB was 72.7%/100%, 63.6%/100%, 54.5%/100%, 27.3%/100%, respectively. The corresponding areas under the curve were 0.864, 0.818, 0.773, and 0.636, successively (**Figure 4B**). These findings further verified the superior performance of MALDI-TOF-MS in the diagnosis of MTB.

## 4 Discussion

The diagnostic value of nucleotide MALDI-TOF-MS for mycobacterial identification based on reference samples or clinical BALF samples from suspected pulmonary mycobacterial infection patients was promising. The high sensitivity, specificity and low LOD of nucleotide MALDI-TOF-MS in mycobacterial detection will greatly improve the positive rate of diagnosis and treatment of TB patients.

There have been studies reporting protein MALDI-TOF MS for the identification of NTM isolates has a concordance rate of 94% with the reference method (Rodríguez-Sánchez et al., 2015; Body et al., 2018). Through optimization of sample inactivation and protein extraction, the accuracy of MALDI-TOF MS in mycobacterial identification was improved to 93.9% (Luo et al., 2018). Absolutely, optimized protein extraction protocol can greatly improve the detection rate (Rodriguez-Temporal et al., 2018). Nucleotide MALDI-TOF MS allows DNA extraction directly from specimens instead of the protein extraction from isolates, which cost a period time to obtain isolates by culture for clinical application. In this study, the concordance rate of the optimized nucleic MALDI-TOF MS for MTB and NTM identification was 97.22 %. A systematic review of 25 studies on Xpert and LAMP for the diagnosis of pulmonary TB using culture as the reference method showed the pooled sensitivity and specificity were 89% and 98%, 93% and 94%, respectively (Yan et al., 2016). In 2013, WHO revised the diagnostic criteria for TB, recommending that the positive detection results of Xpert and LAMP-TB technology should be regarded as positive bacteriological tests, which could be used as the basis for the diagnosis of pathogen-positive TB (Sha, 2021). In this study, we found that the performance characteristics of nucleic MALDI-TOF MS and Xpert were similar or even better, with sensitivity and specificity of 72.7% and 100%, 63.6% and 100%, respectively, suggesting the nucleic MALDI-TOF MS may be a potential assay for mycobacterial identification.

?>According to the WHO Global Tuberculosis Report, the global positive rate of pathogens was 58% in 2020 (Ren et al., 2020). In clinical validation, 18 out of 33 (54.5%) patients who were clinically diagnosed with TB were positive with etiological method. Combined with molecular biological methods, 24/33 (72.7%) cases of TB were found to have etiological basis. Unfortunately, there were still 8 cases clinically diagnosed with TB infection but negative for the four methods we used. In this study, the molecular methods including Xpert and MALDI-TOF MS improved the diagnostic efficiency of confirmed TB cases to 6/33 (18.2%). The positive rates of MALDI-TOF-MS, Xpert, culture and AFS in BALF of patients diagnosed with TB infection were 72.7%, 63.6%, 54.5%, and 27.3%, respectively, similar to 54.6%, 50.4%, 32% of Xpert, culture and AFS in patients with suspected pulmonary mycobacterial infection (Shen et al., 2020). In clinical validation, we found that nucleotide MALDI-TOF-MS showed the largest AUC in detecting MTB compared with other methods, indicating a superior performance of nucleotide MALDI-TOF-MS in the diagnosis of MTB. The nucleotide MALDI-TOF-MS has been used for identification, typing, and drug-resistance detection of pathogens, with the advantages of shorter turn-around time, higher throughput, and lower cost than traditional phenotypic drug susceptibility test (Trembizki et al., 2014). Through the self-built mass spectrometry analysis platform, the automated analysis of batch results can be carried out, greatly improving the speed of test reporting. In terms of mycobacterial identification, the nucleotide MALDI-TOF-MS with the analysis process optimized in this study improved the accuracy to 97.22%.Wu et al. applied nucleic MALDI-TOF-MS to evaluate TB drug resistance, and found that nucleotide MALDI-TOF-MS could be a promising tool for rapid detection of MTB drugs (Wu et al., 2022). TB drug resistance is an important research issue that needs to be addressed today. In the future, we will further explore the correlation between clinical phenotypic outcomes and molecular genotypes.

Our study has some limitations. First, the sample size used for clinical validation was small, leading to fewer patients in the negative group when assessing specificity. Second, we did not analyze the detection of gene resistance against TB by MALDI-TOF MS. Third, we only analyzed the value of MALDI-TOF MS in the detection of mycobacteria in BALF samples, but not other sample types, such as sputum, tissue, cerebrospinal fluid, pleural effusion, etc., which limited the establishment of evidence-based medicine for the diagnostic value of MALDI-TOF MS in extrapulmonary TB. Additionally, due to the low incidence of NTM disease, there were not enough specimens containing NTM for analysis when the clinical validation samples were included. In the future, we will enroll large samples to evaluate the specificity of nucleic MALDI-TOF MS and investigate the difference between the drug resistance genotypes by MALDI-TOF MS and phenotypes.

In summary, optimized nucleotide MALDI-TOF-MS has satisfactory sensitivity, specificity and low LOD in the identification of mycobacteria, which may serve as a potential assay for mycobacterial identification.

## Data availability statement

The original contributions presented in the study are included in the article/**Supplementary Material**. Further inquiries can be directed to the corresponding authors.

## Ethics statement

The studies involving human participants were reviewed and approved by medical committee of Affiliated Hangzhou Chest Hospital, Zhejiang University School of Medicine (No.2022-75). The patients/participants provided their written informed consent to participate in this study.

## Author contributions

BL, CZ participated in writing the manuscript; LS, HD, YS conducted the study design; SC, QZ built the detection platform and optimize the process; LZ, QQ, TM, HW, MQ provided clinical information and case data; CS, QC were in charge of the whole research project. All authors contributed to the article and approved the submitted version.

## Funding

This work was supported by project of Zhejiang Provincial Health Commission (NO.2023KY968).

## Acknowledgments

We thank Furong Du for polishing the article, Jing Liu, Mengji Yu for analysis of results, Hongli Zhou for image optimization from Nanjing Simcere Diagnostics Co., Ltd.

## Conflict of interest

Authors CZ, HD, SC, QZ, and CS were employed by Jiangsu Simcere Diagnostics Co., Ltd.

The remaining authors declare that the research was conducted in the absence of any commercial or financial relationships that could be construed as a potential conflict of interest.

## Publisher’s note

All claims expressed in this article are solely those of the authors and do not necessarily represent those of their affiliated organizations, or those of the publisher, the editors and the reviewers. Any product that may be evaluated in this article, or claim that may be made by its manufacturer, is not guaranteed or endorsed by the publisher.
