# Supplementary material for: Performance evaluation and clinical validation of optimized nucleotide MALDI-TOF-MS for mycobacterial identification
Source: Front Cell Infect Microbiol. 2022 Dec 2;12:1079184. doi: 10.3389/fcimb.2022.1079184 (PMC9755490; doi:10.3389/fcimb.2022.1079184)
Supplement: Supplementary file 1 [file Table_1.docx]

**Supplementary Table 1.** The verification results of mNGS in BALF samples

| Order | [Numbered list](javascript:;) | Mycobacterium detection | Reads |
| --- | --- | --- | --- |
| 1 | SPU38060032A1D1 | Mycobacterium tuberculosis | 719 |
| 2 | SPU24044335A1D1 | Mycobacterium tuberculosis | 43 |
| 3 | SPU29030547P1D1 | Mycobacterium tuberculosis | 213 |
| 4 | SPU50034398P1D1 | Mycobacterium tuberculosis | 170 |
| 5 | SPU86052135A1D1 | Mycobacterium tuberculosis | 76194 |
| 6 | SCT77064840N1D1 | Mycobacterium tuberculosis | 3185 |
| 7 | tSPU62040030A1D1 | Mycobacterium tuberculosis | 106 |
| 8 | SPU40054756P1D1 | Mycobacterium tuberculosis | 6889 |
| 9 | SPU95077277A1D1 | Mycobacterium tuberculosis | 661 |
| 10 | SPU81074566A1D1 | Mycobacterium tuberculosis | 645 |
| 11 | SPU22042129A1D1 | Mycobacterium tuberculosis | 37626 |
| 12 | SPU80046400A1D1 | Mycobacterium tuberculosis | 19923 |
| 13 | SPU17058970P1D1 | Mycobacterium tuberculosis | 524 |
| 14 | SPU68063687A1T1 | Mycobacterium tuberculosis, Mycobacterium abscess (target) | 19 |
| 15 | SPU40034400A1D1 | Mycobacterium abscess | 10 |
| 16 | SPU54034395A1D1 | Mycobacterium abscess | 4 |
| 17 | SPU37047859A1D1 | Mycobacterium abscess | 7 |
| 18 | SPU28054224A1D1 | Mycobacterium abscess | 6 |
| 19 | SPU87043041A1D1 | Mycobacterium abscess | 4 |
| 20 | SPU66071361A1D1 | Mycobacterium abscess | 4 |
| 21 | SPU51043243A1D1 | Mycobacterium abscess | 3 |
| 22 | SPU22055906P1D1 | Mycobacterium abscess | 5307 |
| 23 | SPU75057296A1D1 | Mycobacterium tortoise | 5 |
| 24 | SPU22045410A1D1 | Mycobacterium tortoise | 8 |
| 25 | SPU93034482A1D1 | Mycobacterium tortoise | 6 |
| 26 | SPU42052173P1D1 | Mycobacterium intracellulare | 6 |
| 27 | SPU87052828A1D1 | Mycobacterium intracellulare | 1831 |
| 28 | SPU74055725A1D1 | Mycobacterium Kansas, Mycobacterium intracellulare | 227/26 |
| 29 | SPU75052596P1D1 | Negative | / |
| 30 | SPU52043233A1D1 | Negative | / |
| 31 | tSPU96033549A1D2 | Negative | / |
| 32 | tSPU55058857A1D1 | Negative | / |
| 33 | tSPU89043229A1D2 | Negative | / |
| 34 | SPU86013303A1D1 | Negative | / |
| 35 | SPU49042273A1D1 | Negative (qPCR: Mycobacterium tuberculosis) | / |
| 36 | SPU84055928A1D1 | Negative (qPCR: Mycobacterium tuberculosis) | / |
| 37 | SPU96046197A1D2 | Negative (qPCR: Mycobacterium tuberculosis) | / |

**Supplementary Table 2**. Positive results of MALDI-TOF-MS in the identification of mycobacterial species

| Order | [Numbered list](javascript:;) | Assay Id | Real Extension rate | Wild_SNR |
| --- | --- | --- | --- | --- |
| 1 | SPU38060032A1D1L1 | MTBC_IS1081 | 0.99925 | 13.2283 |
|  |  | MTBC_IS6110 | 0.96758 | 22.4734 |
| 2 | SPU24044335A1D1L1 | MTBC_IS1081 | 0.97894 | 12.8601 |
|  |  | MTBC_IS6110 | 0.98051 | 21.4622 |
| 3 | SPU29030547P1D1L1 | MTBC_IS1081 | 0.9873 | 21.6407 |
|  |  | MTBC_IS6110 | 0.9696 | 22.916 |
| 4 | SPU49042273A1D1 | MTBC_IS1081 | 0.9889 | 19.953 |
|  |  | MTBC_IS6110 | 0.9346 | 21.6193 |
| 5 | SPU84055928A1D1 | MTBC_IS6110 | 0.98854 | 19.1208 |
|  |  | MTBC_IS1081 | 0.41062 | 4.92373 |
| 6 | SPU50034398P1D1L1 | MTBC_IS6110 | 1 | 17.6083 |
|  |  | MTBC_IS1081 | 1 | 15.5394 |
| 7 | SPU86052135A1D1L1 | MTBC_IS1081 | 1 | 20.4229 |
|  |  | MTBC_IS6110 | 1 | 17.7316 |
| 8 | SPU96046197A1D1L1 | MTBC_IS1081 | 0.96596 | 5.99252 |
|  |  | MTBC_IS6110 | 0.99792 | 25.121 |
| 9 | SCT77064840N1D1L1 | MTBC_IS6110 | 0.99299 | 18.7819 |
|  |  | MTBC_IS1081 | 0.9934 | 18.9982 |
| 10 | tSPU62040030A1D1 | MTBC_IS6110 | 1 | 16.6753 |
|  |  | MTBC_IS1081 | 0.98103 | 9.29613 |
| 11 | SPU40054756P1D1 | MTBC_IS6110 | 0.98266 | 21.6006 |
|  |  | MTBC_IS1081 | 0.99518 | 15.2667 |
| 12 | SPU95077277A1D1 | MTBC_IS6110 | 0.9815 | 21.2961 |
|  |  | MTBC_IS1081 | 0.99424 | 8.69877 |
| 13 | SPU81074566A1D1 | MTBC_IS6110 | 0.97602 | 20.0381 |
|  |  | MTBC_IS1081 | 0.97037 | 12.6402 |
| 14 | SPU22042129A1D1 | MTBC_IS6110 | 0.98024 | 19.9753 |
|  |  | MTBC_IS1081 | 0.98623 | 14.9237 |
| 15 | SPU80046400A1D1 | MTBC_IS1081 | 0.99918 | 8.43778 |
|  |  | MTBC_IS6110 | 0.9699 | 24.3578 |
| 16 | SPU17058970 | MTBC_IS1081 | 0.97872 | 13.9616 |
|  |  | MTBC_IS6110 | 1 | 18.88 |
| 17 | SPU68063687A1T | MTBC_IS1081 | 0.94226 | 9.09826 |
|  |  | MTBC_IS6110 | 0.9753 | 23.2082 |
|  |  | M.mass | 0.49015 | 15.9583 |
| 18 | SPU40034400A1D1L1 | M.abs | 0.95643 | 18.9485 |
| 19 | SPU54034395A1D1L1 | M.abs | 0.95281 | 22.6499 |
| 20 | SPU87043041A1D1 | M.mass | 0.42167 | 15.4468 |
| 21 | SPU66071361A1D1 | M.abs | 0.97933 | 24.6114 |
| 22 | SPU22055906P1D1 | M.mass | 0.235322 | 3.58681 |
| 23 | SPU75057296A1D1L1 | M.che | 0.39905 | 6.51412 |
| 24 | SPU22045410 | M.che | 0.99712 | 12.9204 |
| 25 | SPU93034482A1D1L1 | M.che | 0.5892 | 13.9315 |
| 26 | SPU42052173P1D1L1 | M.intr_2 | 0.84342 | 18.1621 |
| 27 | SPU87052828A1D1 | M.intr_2 | 0.79667 | 19.737 |
| 28 | SPU74055725A1D1 | M.intr_2 | 0.74116 | 13.0135 |
|  |  | M.kan | 0.42036 | 6.32662 |
| 29 | TB1 | MTBC_IS1081 | 0.98087 | 9.28315 |
|  |  | MTBC_IS6110 | 1 | 18.6235 |
| 30 | TB2 | MTBC_IS1081 | 0.98855 | 16.9949 |
|  |  | MTBC_IS6110 | 1 | 17.7562 |
| 31 | TB3 | MTBC_IS1081 | 0.99131 | 20.4697 |
|  |  | MTBC_IS6110 | 1 | 18.1387 |
| 32 | TB4 | MTBC_IS6110 | 1 | 17.9428 |
|  |  | MTBC_IS1081 | 0.98499 | 20.6594 |
| 33 | TB5 | MTBC_IS6110 | 1 | 14.8759 |
|  |  | MTBC_IS1081 | 0.9888 | 12.1739 |
| 34 | TB6 | MTBC_IS1081 | 0.98253 | 9.74476 |
|  |  | MTBC_IS6110 | 1 | 18.2798 |
| 35 | TB7 | MTBC_IS1081 | 0.97948 | 14.6392 |
|  |  | MTBC_IS6110 | 1 | 18.3685 |
| 36 | 1#-1 | MTBC_IS1081 | 0.99989 | 11.9301 |
|  |  | MTBC_IS6110 | 1 | 16.8123 |
| 37 | S1 | MTBC_IS1081 | 1 | 8.06305 |
|  |  | MTBC_IS6110 | 1 | 15.3903 |
| 38 | S2 | MTBC_IS6110 | 0.83535 | 21.3047 |
| 39 | S3 | MTBC_IS6110 | 1 | 13.7617 |
|  |  | MTBC_IS1081 | 1 | 7.19631 |
| 40 | S4 | MTBC_IS1081 | 1 | 9.99485 |
|  |  | MTBC_IS6110 | 1 | 24.753 |
| 41 | S5 | MTBC_IS1081 | 0.98487 | 10.5297 |
|  |  | MTBC_IS6110 | 0.90713 | 22.9698 |
| 42 | S6 | MTBC_IS1081 | 0.99505 | 25.3773 |
|  |  | MTBC_IS6110 | 1 | 20.2514 |
| 43 | S7 | MTBC_IS1081 | 0.98666 | 21.9884 |
|  |  | MTBC_IS6110 | 0.98877 | 20.0837 |
| 44 | S8 | MTBC_IS1081 | 0.99352 | 16.4023 |
|  |  | MTBC_IS6110 | 1 | 16.1323 |
| 45 | NTM17 | M.abs | 0.94654 | 16.1309 |
|  |  | M.abc | 0.4671 | 17.9717 |
| 46 | 3#-1 | M.abc | 0.34391 | 10.65 |
|  |  | M.abs | 0.9754 | 28.0361 |
| 47 | 2#-1 | M.kan | 0.51575 | 10.4217 |
| 48 | 3#-2 | M.che | 1 | 13.5942 |
| 49 | 6#-1 | MTBC_IS1081 | 1 | 8.06418 |
|  |  | M.shim | 0.96119 | 18.9749 |
| 50 | 9#-1 | M.intr_2 | 0.53664 | 16.3027 |
| 51 | 10#-1 | MTBC_IS1081 | 0.977736 | 12.8146 |
|  |  | MTBC_IS6110 | 1 | 18.8975 |
|  |  | M.sim | 0.415849 | 10.0155 |
| 52 | NTM16 | M.kan | 0.41447 | 7.6499 |
| 53 | NTM29 | M.intr_2 | 0.38851 | 10.19 |
| 54 | NTM21 | M.avc_1311 | 1 | 20.6319 |
|  |  | M.avc_901 | 0.84268 | 25.4553 |
| 55 | NTM20 | M.smeg | 1 | 35.4512 |
| 56 | NTM22 | M.for | 0.91416 | 21.9314 |
|  |  | M.abs | 0.99145 | 17.6714 |
| 57 | NTM25 | M.xen | 0.88727 | 18.5152 |
| 58 | NTM26 | M.scr | 0.90398 | 17.7952 |
| 59 | NTM30 | M.terr | 0.96926 | 27.6831 |
| 60 | NTM32 | M.gor | 1 | 39.9943 |
| 61 | NTM39 | M.gor | 0.33838 | 9.86861 |
|  |  | M.mar_erp | 1 | 33.9912 |
| 62 | NTM19 | M.cela | 0.97706 | 13.2762 |
| 63 | NTM23 | M.asia | 1 | 14.8527 |
| 64 | NTM31 | M.pere | 0.30092 | 21.9562 |
| 65 | NTM36 | M.intr_2 | 0.71314 | 14.7276 |
|  |  | M.chim | 0.37756 | 7.62851 |
| 66 | M.cela | M.cela | 0.95948 | 15.6042 |
| 67 | M.asia | M.asia | 0.9803 | 24.7823 |
| 68 | M.ulce | M.ulce | 0.85113 | 28.2787 |
| 69 | M.gast | M.gast | 1 | 11.9403 |
| 70 | M.pere | M.pere | 0.46364 | 19.298 |
| 71 | M.sept | M.sept | 0.99642 | 38.8255 |
| 72 | M.chim | M.chim | 0.28464 | 3.71593 |
| 73 | M.mass | M.mass | 0.99622 | 38.442 |
| 74 | M.gena | M.gena | 1 | 52.9408 |
| 75 | P1 | MTBC_IS6110 | 0.82355 | 21.3731 |
| 76 | P2 | MTBC_IS1081 | 0.97462 | 10.6438 |
|  |  | MTBC_IS6110 | 0.96371 | 19.1356 |
| 77 | P3 | MTBC_IS1081 | 0.77622 | 10.9652 |
|  |  | MTBC_IS6110 | 0.82397 | 15.8687 |
| 78 | P4 | MTBC_IS1081 | 0.88991 | 6.88132 |
|  |  | MTBC_IS6110 | 0.77322 | 13.9249 |
| 79 | P5 | MTBC_IS1081 | 0.96286 | 11.6084 |
|  |  | MTBC_IS6110 | 0.70776 | 24.0053 |
| 80 | P6 | MTBC_IS6110 | 0.82355 | 21.3731 |
| 81 | P7 | MTBC_IS1081 | 0.97675 | 10.5754 |
|  |  | MTBC_IS6110 | 0.96507 | 19.1099 |
| 82 | P8 | MTBC_IS1081 | 0.7829 | 10.8851 |
|  |  | MTBC_IS6110 | 0.82317 | 15.8427 |
| 83 | P9 | MTBC_IS1081 | 0.39593 | 6.8042 |
|  |  | MTBC_IS6110 | 0.90242 | 14.2676 |
| 84 | P10 | MTBC_IS1081 | 0.98771 | 17.651 |
|  |  | MTBC_IS6110 | 0.92562 | 15.8781 |
| 85 | P11 | MTBC_IS6110 | 0.99203 | 18.3779 |
|  |  | MTBC_IS1081 | 0.99037 | 19.3986 |
| 86 | P12 | MTBC_IS1081 | 0.98452 | 14.6213 |
|  |  | MTBC_IS6110 | 1 | 17.8991 |
| 87 | P13 | MTBC_IS1081 | 0.99096 | 23.0978 |
|  |  | MTBC_IS6110 | 1 | 18.0101 |
| 88 | P14 | MTBC_IS1081 | 0.979 | 11.6058 |
|  |  | MTBC_IS6110 | 1 | 11.0439 |
| 89 | P15 | MTBC_IS1081 | 0.98632 | 15.9369 |
|  |  | MTBC_IS6110 | 0.98134 | 18.2162 |
| 90 | N1 | M.avc_1311 | 0.66941 | 18.266 |
|  |  | M.avc_901 | 0.48738 | 11.5865 |
| 91 | N2 | M.terr | 0.68695 | 20.8062 |
| 92 | N3 | M.shim | 0.34008 | 8.92899 |
| 93 | N5 | M.asia | 0.99823 | 21.4914 |
| 94 | N9 | M.for | 0.73722 | 17.1873 |
